# Personal risk factors and types of sport associated with drive for thinness and drive for muscularity in NextGen athletes

**DOI:** 10.3389/fnut.2024.1392064

**Published:** 2024-06-10

**Authors:** Juliette Maurin, Sophie Labossière, Lara Pomerleau-Fontaine, Véronique Boudreault, Sophie Brassard, Jacinthe Dion, Natalie Durand-Bush, Sylvie Parent, Amélie Soulard

**Affiliations:** ^1^Department of Psychology, Faculty of Humanities, Université de Sherbrooke, Sherbrooke, QC, Canada; ^2^Research Chair in Security and Integrity in Sport, Université Laval, Quebec City, QC, Canada; ^3^Department of Physical Education, Faculty of Education, Université Laval, Quebec City, QC, Canada; ^4^Department of Psychoeducation, Faculty of Education, Université de Sherbrooke, Sherbrooke, QC, Canada; ^5^Faculty of Human Kinetics, Université de Sherbrooke, Sherbrooke, QC, Canada; ^6^Department of Psychology, Université du Québec à Trois-Rivières, Trois-Rivières, QC, Canada; ^7^School of Human Kinetics, Faculty of Health Sciences, University of Ottawa, Ottawa, ON, Canada; ^8^International Research Network on Violence and Integrity in Sport (IRNOVIS), Antwerp, Belgium; ^9^Interdisciplinary Research Center on Intimate Relationship Problem and Sexual Abuse (CRIPCAS), Montreal, QC, Canada; ^10^Équipe Violence Sexuelle et Santé (ÉVISSA), Université du Québec à Montréal, Montreal, QC, Canada

**Keywords:** NextGen athletes, eating disorder symptoms, disordered eating, developing athletes, performance, body image, youth elite athletes

## Abstract

**Introduction:**

Professional and student-athletes are at risk of developing symptoms of eating disorders (ED), including drive for thinness and muscularity due to personal risk factors (e.g., low self-esteem) and sport-specific characteristics (e.g., sport requirements). However, limited studies have focused on ED symptoms among NextGen athletes (identified in Canada as *élite* or *relève*) who compete at the provincial, national, and international levels but are not yet part of national teams. As such, they have access to fewer financial resources and support from their sport federations, which can create additional stress for these athletes. The present study aimed to identify personal risk factors and types of sports associated with (a) drive for thinness and (b) drive for muscularity in NextGen athletes.

**Methods:**

These athletes (*n* = 254) aged between 14 and 25 years old completed an online questionnaire. Perfectionism, self-esteem in sport, drive for thinness, and drive for muscularity were, respectively, assessed by the Multidimensional Inventory of Perfectionism in Sport, the Sport State Self-Esteem Scale, the Eating Attitude Test-26, and the Drive for Muscularity Scale. Other personal risk factor (e.g., athletic status) were measured using in-house questions. Each personal risk factor was included in two multiple linear regressions, respectively, to determine which factors were most associated with drive for thinness and drive for muscularity.

**Results:**

Analyses revealed that perfectionist aspirations during training were linked to a stronger desire for thinness. In addition, not being in school or not having a job were also linked to a stronger desire for thinness. Several variables were found to be associated with a greater desire for muscularity: being a male athlete, playing team sport, weight category sport or endurance sport (as opposed to esthetic sport), having elite status, being less satisfied with one’s current sporting performance and having higher perfectionist aspirations during training.

**Discussion:**

This study offers initial insights into the factors influencing EDs among NextGen athletes, which provides a foundation for deeper exploration and the creation or modification of targeted interventions. These findings can guide sport organizations in creating guidelines and programs to better support the next generation of national athletes (e.g., create programs to help athletes maintain investments outside).

## Introduction

Athletes are at risk of developing eating disorders (EDs) (1). Up to 32% of male athletes and 45% of female athletes present symptoms of EDs (2, 3). In adolescent athletes, the study by Giel et al. (4) showed ED prevalence rates of 31% in female athletes and 14% in male athletes. Defined as a “persistent disturbance of eating or eating-related behavior that results in the altered consumption or absorption of food and that significantly impairs physical health or psychosocial functioning” [(5), p. 329], EDs include drive for thinness and drive for muscularity. Drive for muscularity refers to attitudes and behaviors aimed at increasing muscularity (6), while drive for thinness is associated with the desire to have a slimmer body (7). This category of mental health disorders is multideterminant in that several biological, psychological, socio-cultural or environmental factors influence their development [e.g., (1, 8)]. Numerous scientific studies of professional athletes and student-athletes have identified the main factors associated with EDs [e.g., (1)].

The literature shows that several personal factors such as low self-esteem [e.g., (1, 9, 10)] and a high level of perfectionism [e.g., (1, 9, 11)] may play an important role in the development of EDs among athletes. Indeed, some authors demonstrate that low self-esteem and high levels of perfectionism are associated with poorer mental health in elite athletes (12–15), and these variables would be considered risk factors for eating disorders in the literature on elite athletes (1). It seems that taking control of diet and body shape could enable individuals with EDs to boost their self-esteem (16). Further, perfectionism is expressed through self-evaluation of the pursuit and attainment of demanding standards in the areas of diet, shape and body weight (17). Given the potential risk of these variables in the development of eating disorders, it seems essential to assess them in order to better understand eating disorders in a population of high-level athletes. Balancing an athletic career with academic studies or employment can also represent a significant stressor for athletes, potentially making them more vulnerable to the development of ED symptoms (1, 18, 19). Other personal factors such as being female, undergoing identity and pubertal development, belonging to the LGBTQ+ community, having a lower level of education, early sport specialization and competing at a high level are also associated with EDs (1, 20–24). For example, the period of puberty experienced during adolescence involves numerous bodily changes that can put adolescent athletes at increased risk of developing EDs (1). In addition to going through adolescence, some athletes undergo a major transition during their sports career when they leave their family home to move closer to their national team training center, potentially making them more vulnerable to developing EDs (1). Although the literature identifies several personal risk factors related to EDs, it seems that certain characteristics linked to the type of sport practiced by athletes may play a role in the development of such disorders (9).

The practice of certain types of sports [e.g., esthetic and weight-category sports; (1)] as well as the athlete’s position in the sport [e.g., striker; (25)] is known to increase the risk of developing ED symptoms. Specifically, some sports may promote drive for thinness behaviors and attitudes in order to enhance athletic performance [e.g., esthetic sports; (1, 26)]. For example, Mancine et al. (26) showed that, depending on the demands of different sports (e.g., performance judged based on esthetics), the risks of developing drive for thinness behaviors and attitudes vary and appear to be higher in esthetic sports. Other sports (e.g., weight-category sports) may encourage the development of significant muscularity (27). For example, Sundgot-Borgen and Garthe (28) reported that athletes in weight-category sports (e.g., boxing) tend to strive to develop muscularity while maintaining a low body weight. Regarding the athlete’s position, Compte et al. (25) demonstrated that the position practiced in sport (e.g., attack or defense in some team sports) can influence drive for thinness behaviors and attitudes, depending on the associated athletic demands. Thus, depending on the demands of their sport or their position within it, athletes may develop behaviors and attitudes oriented not only toward the drive for thinness, but also toward the drive for muscularity (29, 30).

To our knowledge, current data concerning EDs in athletes have been collected mainly from Olympic and Paralympic athletes and student-athletes in Europe, Australia and the United States [e.g., (3, 6, 31)]. The reality of Canadian NextGen athletes who compete at regional, provincial, national and international levels without being part of national teams, is still relatively unknown, despite the need for prevention and early intervention after the first symptoms of EDs appear (32). NextGen athletes face unique stressors including lack of funding opportunities, less support from sport organizations, and uncertainty over the possibility of securing a place on the national team (33), in a context where many of the athletes are at crucial periods in their development. Some may also be enrolled in studies or may hold a job in parallel with their sporting career, which can make it difficult to reconcile these multiple commitments. In light of these findings, Purcell et al. (32) published an editorial calling on researchers to further study the mental health of NextGen athletes. In particular, the reality of NextGen athletes in relation to EDs must be investigated in order to paint an accurate and representative portrait of this problem within this population. The influence of several factors identified in professional athletes and student-athletes (e.g., type of sport, age, and perfectionism) might be exacerbated in NextGen athletes due to their precarious context.

Furthermore, most studies of EDs have focused on typically feminine representations (drive for thinness behaviors and attitudes) of these disorders (34). Given that measurement instruments that assess the presence or absence of ED have been constructed almost exclusively with female populations, males are currently underrepresented in ED studies (34). As the male body ideal is geared more toward a muscular body, EDs in men would be more focused on the drive for greater muscularity (34), although this issue may also affect some female athletes (35). Further, it seems that the drive for thinness also affects some male athletes (2, 36, 37). For example, Karrer et al. (2) found that up to 32.5% of male athletes demonstrated drive for thinness behaviors and attitudes. The present study therefore took into account both drive for thinness (typically female representation) and drive for muscularity (typically male representation) behaviors and attitudes in male and female athletes, in order to obtain a holistic picture of ED issues.

Thus, the present study aimed to identify personal risk factors and types of sport associated with ED symptoms reported by NextGen athletes. More specifically, this study had two objectives: (1) to identify the personal risk factors (sex at birth, age, athletic status, sexual orientation, self-esteem, perfectionism, occupation, age of start of competition in main sport, and remoteness from family for training) and types of sport (team sport, endurance sport, weight-category sport, technical sport, and esthetic sport) associated with drive for thinness and (2) to identify the personal risk factors and types of sport associated with drive for muscularity in NextGen athletes. According to the literature on risk factors for EDs in sport [e.g., (1, 2)], we hypothesize that personal risk factors (e.g., high level of perfectionism, low self-esteem) will be associated with more ED symptoms.

## Materials and methods

### Participants and recruitment procedure

The recruitment process (April–July 2022) included two strategies and was facilitated by the collaboration of multiple Canadian sporting organizations (e.g., regional centers, and provincial sport federations). First, the ALEO foundation (Foundation offering grants and support services to student-athletes) sent an email to francophone NextGen athletes via their provincial sport organization inviting them to complete an online survey. Second, a social media page was created specifically for the current study where advertisements were crafted to promote recruitment among the target population, which is highly active on social media. Participants who were interested had to contact the researchers via an email address. The survey link was then shared with the French-Canadian NextGen athletes who were aged between 14 and 25 years. The sample was delimited to francophone athletes given their under-representation in Canadian sport psychology research. Participants had a chance to win one of two pairs of wireless headphones. This study was approved by the Research Ethics Committee Education and Social Sciences of the University of Sherbrooke (2021–2809).

Considering empirical guidelines regarding NextGen athletes (38), the age of athletes spanned from 14 to 25 years. In Canada, NextGen athletes compete at the provincial, national, or international level but are not part of a national team. They are not recognized by a National Sport Organization (NSO), but rather by a Provincial Sport Organization (PSO). Based on specific criteria, they can be categorized into two different groups: (a) *élite* athletes or (b) *relève* athletes. Although criteria for identifying athletes as *élite* or *relève* change across PSOs (39), athletes who are identified as *élite* generally compete at a higher level, and the demands of their sport are also heightened. Athletes from the Quebec Major Junior Hockey League were also recruited because they share a similar profile to NextGen athletes. Other athletes were excluded from the current study because they were beyond the delimited age range, or competed at a different level (e.g., regional).

Overall, 769 athletes accessed the online questionnaire using LimeSurvey software. After removing the participants who did not provide their consent, complete the questionnaire, or meet the inclusion criteria, the final sample included 254 NextGen athletes (35.5% men and 64.5% women) with an average age of 18.08 (SD = 2.34). 91.7% of participants are heterosexual, and the majority are in secondary school (35.8%) or have a secondary school diploma (46.1%). In terms of types of sport, 31.1% (*n* = 79) of participants take part in team sports, 30.7% (*n* = 78) in endurance sports, 11.8% (*n* = 30) in esthetic sports, 11.8% (*n* = 30) in weight category sports and 14.6% (*n* = 37) in technical sports. 51.2% of participants identified the national level as their highest level of participation in competition, while 24.8% identified the international level. The remaining participants identified regional (0.4%), provincial (11.8%) or North American (11.8%) levels (see Supplementary material for sociodemographic characteristics).

### Measures

#### Sociodemographic questions

Participants completed a series of sociodemographic questions about their age, sex assigned at birth (female/male/intersex), sexual orientation (heterosexual/homosexual/bisexual/other), athletic status (*relève*/*élite*), employment (yes/no), being in school (yes/no), and the age they started competing in their sport. Among these variables, sex at birth was dichotomized (male/female) due to an absence of entries for the intersex category. Sexual orientation was also dichotomized (heterosexual/other sexual orientation), due to a lack of entries for bisexual, homosexual, and other sexual orientation. Athletes were also invited to indicate the principal sport in which they were competing. These sports were then categorized into five different types (team, endurance, technical, weight-category, and esthetic) based on the literature [e.g., (40)] and following meetings with a committee of experts. The different characteristics associated with each type of sport are presented in Supplementary material.

##### Eating attitude test-26

To measure athletes’ drive for thinness, the self-reported French version (41) from the original EAT-26 questionnaire (42) was used. The participants answered 26 questions with a six points Likert Scale (Never = 0, Rarely = 0, Sometimes = 0, Often = 1, Usually = 2, and Always = 3) and the total score varies between 0 and 78. Scores of 20 and above indicate clinical symptoms for drive for thinness (41, 42). The validated French EAT-26 questionnaire has shown good psychometric characteristics (41), and good internal consistency for the current study (Cronbach’s alpha = 0.879; McDonald’s omega = 0.892).

##### Drive for muscularity scale-French

To measure athletes’ drive for muscularity, the French 9-item version (43) of the original DMS questionnaire (44) was used [for more information about this French 9-item version of the DMS, see the validation article by Chaba et al. (43)]. Participants answered each item with a Likert scale ranging from 1 (not at all) to 6 (absolutely), with a total score varying between 9 and 54. In the current study, the DFM-FR has shown an acceptable to good internal consistency (Cronbach’s alpha = 0.788; McDonald’s omega = 0.825).

##### Sport-state self-esteem scale

The S-SSES was used to assess athletes’ level of self-esteem (45). Most specifically, the French version of the S-SSES (46), which has previously shown adequate validity and reliability (46), was used for the current study, Participants answered 10 items through a Likert scale (1 = completely agree, 6 = completely disagree). This instrument also includes two subscales: Satisfaction with Current Sport Performances (score 1–5) and Perceived Athletic Competence (score 1–5). For the current study, the internal consistency ranged from questionable to good: Satisfaction with Current Sport Performance (Cronbach’s alpha = 0.875; McDonald’s omega = 0.870), Perceived Athletic Performance (Cronbach’s alpha = 0.650; McDonald’s omega = 0.663), and Global Self-Esteem in sport (Cronbach’s alpha = 0.709; McDonald’s omega = 0.873).

##### Multidimensional inventory of perfectionism in sport

To assess athletes’ perfectionistic aspirations during training, negative reactions to non-perfect performance during training as well as the perception of pressure from parents to be perfect, the MIPS was used, comprising 24 items [each dimension includes eight items; (47)]. The MIPS has shown good factorial validity (48) and high reliability and validity (47). Participants responded to each item on a Likert scale, ranging from 1 (never) to 6 (always). The overall perfectionism score ranged from 1 to 6. For the present study, all subscales demonstrated good to excellent internal consistency: pressure from parents (Cronbach’s alpha =0.945; McDonald’s omega = 0.945), perfectionistic aspirations during training (Cronbach’s alpha = 0.958; McDonald’s omega = 0.959), negative reactions to non-perfect performance during training (Cronbach’s alpha = 0.915; McDonald’s omega = 0.917), striving for perfection (Cronbach’s alpha = 0.935; McDonald’s omega = 0.935), negative reactions to imperfection (Cronbach’s alpha = 0.867; McDonald’s omega = 0.869), and overall perfectionism (Cronbach’s alpha = 0.900; McDonald’s omega = 0.888).

#### Analyses

All analyses were performed using SPSS software version 29.0.0.0 (49). The 95% confidence level was selected (*p* ≤ 0.05) to reject the null hypothesis. First, descriptive analyses (frequency, percentage, mean, and standard deviation) and a chi-squared test were carried out to describe the sample. To identify the personal risk factors and types of sport associated with drive for thinness (objective 1) and drive for muscularity (objective 2) in NextGen athletes, two multiple linear regressions were performed. In each of the two regressions, 17 independent variables were included simultaneously: personal variables and types of sport. Drive for thinness and drive for muscularity were the dependent variables in the first and the second regressions, respectively.

A power analysis was performed on a multiple regression sample size calculator (50). It suggested that when running a linear multiple regression with 17 predictors for a *p* value of 0.05, a power of 0.80, and a small-medium effect size of *f*^2^ = 0.09, a sample of 233 participants would be necessary to detect a significant effect.

Before performing these multiple linear regression analyses, correlations between variables were observed to ensure the absence of multicollinearity between independent variables (the correlation matrix between the variables under study is available in Supplementary material). On this basis, some variables were not included in the regressions, as they were too strongly correlated with the other variables of interest [*r ≥* 0.80, (51)], that is, overall sport self-esteem, striving for perfection, negative reactions to imperfection and overall perfectionism. In the regressions, no multicollinearity was observed; tolerances were all greater than 0.05 (52). To ensure the normality of the dependent variable for each level of the independent variables, residual diagnostics were also performed. As the drive for thinness eating attitudes and behaviors did not meet this normality assumption, this variable was log-transformed (*ln + 1*) before being included in the regressions. In order to obtain the most parsimonious models possible, each of the two regressions was rolled again, including only the significant variables.

## Results

Of the total sample, 8.27% of participants reached the clinical threshold of the EAT-26 (measuring drive for thinness behaviors and attitudes). For women, this ratio was 9.15%, while for men it was 6.67%. The Chi-square test did not reveal any significant differences by sex: *X*^2^(1) = 0.47, *p* = 0.33.

Of the 17 variables studied, only two were found to be significantly associated with drive for thinness behaviors and attitudes: perfectionistic aspirations and school and job involvement (Table 1). In the parsimonious model, perfectionistic aspirations during training and occupation (school and job involvement) significantly explained 10.7% of the variance in drive for thinness behaviors and attitudes. More specifically, a higher level of perfectionistic aspirations during training was significantly associated with more of these attitudes and behaviors, while being in school or employed, as well as being in school and employed (compared with not going to school and not being employed) were significantly associated with fewer drive for thinness attitudes and behaviors. Among these variables, some contributed more to the model: Being in school and employed (β = −0.43) was the most significant contributor, followed by being in school or employed (β = −0.38) and perfectionistic aspirations during training (β = 0.30).

**Table 1:** Multiple linear regressions of drive for thinness (*N* = 254).

	Global model		Parsimonious model	
	*b* (SE)	β	*b* (SE)	β
	95% CI		95% CI	
Individual variables				
Sex at birth	0.153 (0.97)	0.100		
	−0.039; 0.345			
Age	0.003 (0.023)	0.010		
	−0.043; 0.049			
Athletic status	0.034 (0.103)	0.023		
	−0.168; 0.237			
Sexual orientation	0.074 (0.169)	0.028		
	−0.259; 0.407			
Satisfaction with current athletic performance	−0.069 (0.061)	−0.097		
	−0.189; 0.051			
Perceived athletic competence	0.045 (0.70)	0.049		
	−0.093; 0.183			
Perfectionistic aspirations during training	0.118 (0.043)^**^	0.202	0.173 (0.035)^***^	0.295
	0.033; 0.203		0.104; 0.242	
Negative reactions to non-perfect performance during training	0.098 (0.059)	0.145		
	−0.018; 0.214			
Pressure from parents to be perfect	−0.006 (0.048)	−0.008		
	−0.100; 0.088			
Occupation^1^				
Being in school or employed	−0.506 (0.239)^*^	−0.345	−0.558 (0.239)^*^	−0.380
	−0.977; −0.034		−1.029; −0.087	
Being in school and employed	−0.622 (0.242)^*^	−0.421	−0.630 (0.241)^**^	−0.427
	−1.099; −0.145		−1.105; −0.156	
Age of start of competition in main sport	−0.021 (0.014)	−0.098		
	−0.049; 0.007			
Remoteness from family for training	0.000 (0.001)	−0.057		
	−0.001; 0.001			
Type of sport^2^				
Team sport	0.059 (0.156)	0.037		
	−0.249; 0.367			
Endurance sport	0.019 (0.158)	0.012		
	−0.293; 0.331			
Weight-category sport	0.278 (0.185)	0.122		
	−0.087; 0.642			
Technical sport	0.058 (0.179)	0.028		
	−0.294; 0.410			
Constant	1.587 (0.594)^*^		1.810 (0.278)^***^	
	0.417; 2.756		1.262; 2.358	
Eating disorders symptoms adjusted *R*^2^	0.126^***^		0.107^***^	

*b*, Unstandardized beta coefficient; SE, Standard error; β, Standardized beta coefficient; CI, Confidence intervals (lower 2.5%; upper 2.5%). The analyses included dummy coded variables. ^1^The first variable is the type of commitment the participants had in parallel with their sport: being neither in school nor employed (reference category), being in school or employed [1 = being in school or employed, 0 = other commitment, and being in school and employed (1 = being in school and employed, 0 = other commitment)]. ^2^The second variable is the category of sport practiced by participants: team sport (1 = team sport, 0 = other sport), endurance sport (1 = endurance sport, 0 = other sport), esthetic sport (reference category), weight-category sport (1 = weight-category sport, 0 = other sport), and technical sport (1 = technical sport, 0 = other sport). ^*^p ≤ 0.050, ^**^p ≤ 0.010, and ^***^p ≤ 0.001.

Of the 17 variables studied, seven were found to be significantly associated with drive for muscularity behaviors and attitudes: sex at birth, athlete status, perfectionistic aspirations, satisfaction with athletic performance, and type of sport (Table 2). In the most parsimonious model, sex at birth, athlete status, satisfaction with current athletic performance, perfectionistic aspirations during training and participation in team, endurance and weight-category sports (compared with esthetic sports) significantly explained 19.0% of the variance in drive for muscularity. More specifically, being male, being a higher level athlete, being less satisfied with current athletic performance, having more perfectionistic aspirations during training and practicing a team, endurance or weight-category sport (vs. an esthetic sport) were more significantly associated with drive for muscularity. The contribution of these variables to the regression model varied: competing in an endurance sport (β = 0.31) was the most significant contributor, followed by competing in a team sport (β = 0.25), sex at birth (β = −0.24), perfectionistic aspirations during training (β = 0.23), satisfaction with current sport performance (β = −0.20), competing in a weight-category sport (β = 0.16), and athlete status (β = 0.15).

**Table 2:** Multiple linear regressions of drive for muscularity (*N* = 254).

	Global model		Parsimonious model	
	*b* (SE)	β	*b* (SE)	β
	95% CI		95% CI	
Individual variables				
Sex at birth	−4.023 (1.016)^***^	−0.244	−3.931 (0.978)^***^	−0.238
	−6.025; −2.021		−5.857; −2.005	
Age	−0.265 (0.243)	−0.079		
	−0.745; 0.214			
Athletic status	2.884 (1.071)^**^	0.182	2.429 (0.948)^*^	0.153
	0.773; 4.995		0.561; 4.298	
Sexual orientation	1.044 (1.763)	0.034		
	−2.428; 4.517			
Satisfaction with current athletic performance	−1.518 (0.635)^*^	−0.199	−1.516 (0.454)^***^	−0.198
	−2.770; −0.267		−2.411; −0.622	
Perceived athletic competence	0.556 (0.732)	0.056		
	−0.886; 1.997			
Perfectionistic aspirations during training	1.304 (0.448)^**^	0.207	1.456 (0.371)^***^	0.231
	0.421; 2.186		0.726; 2.186	
Negative reactions to non-perfect performance during training	0.504 (0.614)	0.069		
	−0.706; 1.713			
Pressure from parents to be perfect	−0.269 (0.497)	−0.035		
	−1.247; 0.710			
Occupation				
Being in school or employed^1^	1.810 (2.495)	0.114		
	−3.104; 6.724			
Being in school and employed^1^	3.043 (2.525)	0.191		
	−1.932; 8.018			
Age of start of competition in main sport	0.153 (0.148)	0.067		
	−0.139; 0.445			
Remoteness from family for training	−0.004 (0.005)	−0.048		
	−0.015; 0.006			
Type of sport^2^				
Team sport	4.246 (1.629)^**^	0.249	4.335 (1.566)^**^	0.254
	1.037; 7.455		1.251; 7.418	
Endurance sport	5.412 (1.652)^***^	0.316	5.375 (1.558)^***^	0.314
	2.157; 8.667		2.305; 8.444	
Weight-category sport	3.879 (1.929)^*^	0.159	3.799 (1.847)^*^	0.155
	0.078; 7.680		0.161; 7.437	
Technical sport	2.952 (1.863)	0.132	3.075 (1.776)	0.137
	−0.717; 6.622		−0.424; 6.574	
Constant	18.466 (6.189)^***^		20.223 (3.924)^***^	
	6.273; 30.658		12.494; 27.951	
Drive for muscularity adjusted *R*^2^	0.181^***^		0.190^***^	

*b*, Unstandardized beta coefficient; SE, Standard error; β, Standardized beta coefficient; CI, Confidence intervals (lower 2.5%; upper 2.5%). The analyses included dummy coded variables. ^1^The first variable is the type of commitment the participants had in parallel with their sport: being neither in school nor employed (reference category), being in school or employed [1 = being in school or employed, 0 = other commitment, and being in school and employed (1 = being in school and employed, 0 = other commitment)]. ^2^The second variable is the category of sport practiced by participants: team sport (1 = team sport, 0 = other sport), endurance sport (1 = endurance sport, 0 = other sport), esthetic sport (reference category), weight-category sport (1 = weight-category sport, 0 = other sport), and technical sport (1 = technical sport, 0 = other sport).

^*^p ≤ 0.050, ^**^p ≤ 0.010, and ^***^p ≤ 0.001.

## Discussion

The aim of the present study was to identify personal risk factors and types of sport associated with drive for thinness and drive for muscularity in francophone NextGen athletes. Overall, results revealed that perfectionistic aspirations were associated with both outcomes. Moreover, not being in school or employed was associated with a stronger drive for thinness. Regarding drive for muscularity, more variables were identified, namely identifying as a male athlete, competing in a sport other than an esthetic or technical sport, having *élite* status, and being less satisfied with current athletic performance. It is important to note that several sociodemographic variables (e.g., age, sexual orientation, age of onset of competition, and having moved away from the family residence to train) did not emerge as significantly associated with ED symptoms, allowing us to only partially confirm our hypothesis. The inclusion of these variables did, however, limit possible spurious associations and thus increased the scientific rigor of the results. The results of this study will be discussed in two sections: (1) personal risk factors associated with the presence of drive for thinness or drive for muscularity, and (2) types of sport associated with the presence of drive for thinness or drive for muscularity.

### Personal risk factors associated with the presence of drive for thinness or drive for muscularity

The results of this study showed that being male was more strongly associated with a drive for muscularity behaviors and attitudes. These results are supported by the current research on the influence of sex on drive for muscularity behaviors and attitudes among athletes (35, 53, 54). Compared with female athletes, male athletes are more likely to aspire to develop muscularity in order to improve their sport performance (35, 54). Moreover, the drive for muscularity enables male athletes to correspond more closely to the male body ideal (muscular body) valued in society (34). In contrast, being female was not significantly associated with any of the dependent variables in the study. This result can be explained by the double standard that female athletes face (55). Several studies show that female athletes indeed aspire to develop muscularity in order to enhance their sporting performance, yet they also seek to match the ideal female body (very slim) valued in society (34, 35, 56). In this study, the findings suggest that male and female NextGen athletes may seek to develop their musculature in order to enhance their performance. However, socially valued body ideals (e.g., muscular body for men, very slim body for women) tend to lead more male athletes than female athletes to demonstrate eating behaviors and attitudes reflecting the drive for muscularity (35). This suggests that NextGen athletes are also affected by social pressures relating to ideals of body image, showing the importance to include interventions prior the national level to better support the needs of these athletes. In turn, this would have the potential to positively influence their long-term involvement and satisfaction in their sports.

Regarding the relationship between perfectionism and ED symptoms, “perfectionistic aspirations during training” was associated more strongly with drive for thinness behaviors and attitudes as well as drive for musuclarity behaviors and attitudes. Although results still seem mixed in the literature regarding the association between perfectionism and EDs (1, 2, 9), the results of the present study seem to align with studies supporting an association between these two variables [e.g., (1)]. Further, the “satisfaction with current athletic performance” dimension in relation to self-esteem was found to be more significantly associated with drive for muscularity behaviors and attitudes. This result is in line with studies showing that a lower level of self-esteem is a risk factor for EDs in athletes (1, 9). It seems that a lack of satisfaction with athletic performance could lead to drive for musuclarity behaviors and attitudes. These results could then be explained by the fact that athletes believe that body modification could enhance their performance (57), which enables them to fulfill their perfectionistic aspirations and achieve greater satisfaction with their performance. Sundgot-Borgen and Garthe (28) explain, in their literature review concerning the body challenges that elite athletes may face, why it is advantageous for many athletes to keep body fat at low levels while developing and maintaining great muscle power and strength to perform well. ED symptoms could thus be perceived as utilitarian (e.g., performance-enhancing tool) in athletes [e.g., (58)], underscoring the importance of changing athletes’ belief system regarding the body image required to achieve excellence. This work on athletes’ values and beliefs could be carried out with a mental performance consultant, which underlines the benefits and importance of mental performance work for athletes (59).

No particular dimension related to self-esteem was found to be significantly associated with a higher level of drive for thinness behaviors and attitudes. This runs counter to the ED literature, which tends to show that low self-esteem is associated more strongly with drive for thinness behaviors and attitudes [e.g., (10)]. The choice of the Sport State Self-Esteem Scale (45) to measure athletes’ self-esteem could provide an explanation for this result. This instrument does not assess athletes’ perception of their body image (body esteem) and does not assess their overall self-esteem, but only their satisfaction with their athletic performance and their perceived athletic skills. Some studies show that low body esteem is a risk factor for the development of ED (60–62). Thus, self-esteem may not have emerged as significantly associated with drive for thinness behaviors and attitudes given the omission of the body esteem dimension. Future studies including a larger number of participants practicing a sport that values thinness (e.g., esthetic sports), could incorporate a dimension on body esteem in order to obtain a holistic portrait of self-esteem in relation to EDs.

Although the perfectionism dimension “perfectionistic aspirations during training” was associated more strongly with ED symptoms, the dimension “negative reactions to non-perfect performance during training” was not significantly associated with more ED symptoms. The present study is among the first to associate this last dimension of perfectionism with ED symptoms, if we limit the comparison to the current literature. This result could be explained by the fact that NextGen athletes may have learned to react positively to their non-perfect performance during training in order to reach a high level of sporting achievement. Thus, achieving non-perfect performance would not necessarily generate major dissatisfaction in NextGen athletes, which could reduce their intention to modify their bodies in relation to this performance.

Another dimension of perfectionism that was not significantly associated with ED symptoms was “parental pressure to be perfect.” This finding diverges from recent studies showing that parental pressure can be a risk factor for the mental health of student-athletes (63) and NextGen athletes (64). In addition, Boudreault et al. (65) demonstrated that the behaviors of athletes’ parents can sometimes be problematic and prompt weight-control behaviors in athletes. The results of our study are more in line with those of Cosh and Tully (66), who observed that the parents of student-athletes provided important emotional (e.g., encouragement), practical (e.g., transportation), and financial (e.g., coverage of travel expenses) support for their children in their athletic careers. Thus, parents may be playing a more supportive role with NextGen athletes, rather than exerting additional pressure. Further studies on the reality of NextGen athletes are needed to delve deeper into the role of parents in athletes’ perfectionistic behaviors in the development of EDs in this population.

The results show that *élite* athlete status, compared to the lower *relève* status, is associated more significantly with drive for muscularity behaviors and attitudes, which is in line with the literature [e.g., (2, 67, 68)]. Bonanséa et al. (67) reported that intensive-level athletes (compared with recreational athletes) are significantly more likely to report body modification behaviors. In the present study, athletes identified as *élite* tend to have higher performance standards than athletes identified as *relève*. Therefore, they likely face greater pressure to optimize their performance, making them more inclined to develop dissatisfaction with their body image [e.g., not finding themselves muscular enough to perform, (67)]. This body dissatisfaction could then lead them to develop behaviors aimed at increasing their muscularity to a greater extent than *relève* athletes (9). These results highlight the importance of adjusting available resources according to athletes’ level of competition and using proactive measures with *relève* athletes to prevent them from developing an unhealthy drive for muscularity at the *élite* level.

Further, having no commitments other than sport (studies or employment) was more strongly associated with drive for thinness behaviors and attitudes among the athletes in the present study. This significant association may be explained by the presence of a strong athletic identity. Thus, the lack of investment outside the athletic career may lead athletes to cultivate their identity solely around their sport, that is, they develop a strong and unique athletic identity (69). Ahlich et al. (70) found that athletic identity is positively associated with ED symptoms: the more exclusive the athletic identity, the more likely athletes are to seek to modify their body so that it also matches their role of athlete, and therefore their identity. Although there is no consensus in the literature on the role of athletic identity in EDs [e.g., (71, 72)], Turton et al. (72) suggested that this variable could influence ED symptoms during periods of transition, in which athletic identity would be challenged. However, given their unique context, the majority of NextGen athletes could be experiencing the transition period of adolescence, in addition to facing the uncertainty of their sporting status (waiting to join national teams), which could exacerbate the questioning of their athletic identity. Therefore, opportunities for NextGen athletes to develop a multidimensional identity, such as discovering diverse interests outside their sport, should be facilitated.

### Types of sport associated with drive for thinness or drive for muscularity

The present study found that the practice of team, endurance and weight-category sports, as opposed to esthetic sports, was significantly associated with the presence of drive for muscularity behaviors and attitudes. These results align with the findings in the literature that athletes in these sports benefit from having greater muscularity (28, 35, 73, 74). In the context of the present study, this result could be explained by the demands of team, endurance, and weight-category sports, where a high percentage of body fat could be considered unfavorable to performance, whereas high muscle strength would be considered advantageous (28). In their book on physical strength and conditioning, Reiss and Prévost (73) affirmed the significant advantages of muscularity for endurance, team, and weight-category sports. For example, these authors mention that greater muscle strength can significantly improve explosiveness and aerobic power, two skills often important in team sports. They also pointed out that developing greater muscular strength can improve running economy and joint stability, which is desirable in certain endurance sports. In weight-category sports, athletes aim to have the lowest weight combined with the greatest muscle strength and power (28). By contrast, in esthetic sports (such as dance, gymnastics and synchronized swimming), performance is judged on esthetics, and a slender body shape is sometimes preferred to meet evaluation criteria and facilitate flexibility (26, 75). Thus, athletes practicing an esthetic sport are not likely to exhibit drive for muscularity behaviors and attitudes in order to maintain a body shape conducive to their performance.

Interestingly, among the different types of sport, endurance sports explained the most variance in drive for muscularity. This result adds nuance to previous studies that found that athletes who practice endurance sports are at risk of exhibiting drive for thinness attitudes and behaviors [e.g., (9, 76)]. Endurance sport athletes appear to exhibit more drive for muscularity attitudes and behaviors compared to athletes who practice esthetic sports, yet the two groups do not differ in their drive for thinness behaviors and attitudes. Endurance sports require specific muscle development to enhance performance (73, 77). NextGen athletes who practice endurance sports may therefore be more inclined to engage in muscular strength development in order to optimize their endurance and efficiency rather than aiming for thinness. They may be motivated by the quest for functional, performance-enhancing muscularity rather than esthetic concerns linked to thinness. Future studies with NextGen athletes are needed to confirm this hypothesis.

In light of the current literature on EDs in esthetic sports, it is surprising to note that in the present study, esthetic sports were not significantly associated with more drive for thinness behaviors and attitudes compared with other types of sports [e.g., (9, 26, 78)]. This result could be explained by the increased support available for athletes practicing esthetic sports (79–81). Recent efforts by esthetic sport organizations to support athletes in practicing safe and healthy sport by guiding them in particular toward healthy eating practices may have contributed to reducing the propensity of esthetic sports athletes to experience drive for thinness behaviors and attitudes, compared with their peers in other sports (79–81). In their review of studies evaluating interventions aimed at preventing EDs in athletes, Bigler and Mabillard (79) showed that the research has largely focused on esthetic sports, considered to be a type of sport more at risk in terms of the development of EDs. These examples show that esthetic sports have received a great deal of attention in research and practice, potentially leading to better support for athletes practicing this type of sport. It is also possible to believe that such support has led to changes in sporting culture, resulting in a modification of body expectations in this type of sport. Also, the present study examined only four esthetic sports (gymnastics, diving, synchronized swimming, and figure skating), in addition to having a relatively small convenience sample, given the number of sport categories considered (between 30 and 79 participants per category), which may have influenced the result and thereby limited its generalizability. Nevertheless, these results might imply that the efforts put in place by various sport organizations have a positive influence on EDs among NextGen athletes. Therefore, it is important to pursue such preventive efforts along with the creation of sporting environments conducive to the mental and physical health of athletes, thus influencing their well-being and continuity in their sport. In addition, further studies could examine ED symptoms by type of sport, in larger samples.

### Overview of the results and recommendations

To our knowledge, this study is the first to look at ED symptoms in francophone NextGen athletes, while considering personal factors and types of sport. It identified personal risk factors associated with drive for thinness and drive for muscularity, as well as certain types of sport (endurance sports, weight-category sports, and team sports) associated with drive for muscularity in this population. Interestingly, more of the study variables were found to be more significantly associated with a drive for muscularity than a drive for thinness. A recent position statement on ED symptoms in sports (1) shows that a multitude of factors can influence the development of such symptoms. For example, some authors [e.g., (65, 82)] asserted that the coach-athlete relationship plays a role in EDs. This could be an important factor to consider with NextGen athletes, for whom the coach is often a very present and significant figure (83). Further, in line with the etiological model of ED proposed by Petrie and Greenleaf (84), general societal pressures, in addition to the pressures generated by the culture of sport, appear to play a major role in the development of ED symptoms. Future studies could also assess whether offering athletes mental health training in their sporting environment could help reduce their ED symptoms. Thus, a significant part of the variance in drive for thinness and drive for muscularity remains to be explained. Further research into EDs in NextGen athletes is therefore needed to deepen our understanding of the factors contributing to the development of drive for thinness and drive for muscularity behaviors and attitudes in this population. Furthermore, the development of knowledge concerning the mechanisms underlying the drive of muscularity and thinness could be the subject of future quantitative studies, providing a deeper understanding of the risk factors associated with EDs.

In the present study, 8.27% of participants displayed eating attitudes and behaviors oriented toward the drive for thinness (6.67% in men and 9.15% in women, but no significant sex difference). This rate seems relatively low compared with those reported in the literature among elite athletes, which range from 0 to 32% among male athletes and 6 to 45% among female athletes (1, 2). These differences can be explained in several ways. First, a significant proportion of study participants (31.10%) play team sports, in which body shape and weight are less of a determinant of performance [e.g., (85)]. Second, the tool used to assess the presence or absence of drive for thinness behaviors and attitudes [EAT-26, (42)] was not developed specifically for athletes, which may limit its ability to accurately assess the prevalence of drive for thinness behaviors and attitudes in this population (86). Third, given that high-level athletes are at greater risk of reporting EDs (23), some athletes in the study may not have developed clinical forms of EDs yet, because they are not yet competing at the highest sporting levels (e.g., on national teams). Qualitative studies are needed to better understand the lower rate of drive for thinness behaviors and attitudes, and the lack of significant sex difference observed among NextGen athletes.

### Clinical implications

From a clinical standpoint, the results of the present study highlight the importance of intervening on the level of perfectionism of NextGen athletes and the performance demands of their sport. The prevention and treatment of EDs in NextGen athletes could be facilitated by changes in the athletes’ values and beliefs, particularly regarding the bodily requirements to perform (87). For example, creating an environment where the emphasis is on athletes’ effort, aspirations, and choices rather than their body image and performance outcome has the potential to foster the athletic development of NextGen athletes, while maintaining healthy and caring eating habits. In addition, this study highlights the importance of raising awareness among sport stakeholders of the risks of athletes developing not only drive for thinness, but also drive for muscularity, which can be detrimental to their well-being, mental health and their athletic performance. Finally, the results suggest that encouraging athletes to maintain an investment, interests and involvement outside sport could contribute positively to their athletic performance, mental health and personal development in other life areas (88). Thus, the results of this study could be used for educational purposes by being integrated into mental health training modules for coaches, mental performance consultants, or sport leaders. These professionals would consequently be better equipped to support athletes in their sporting development while considering the risks of developing EDs. Such training would not only help to develop their knowledge regarding mental health and mental performance, but also to prevent mental health problems such as ED symptoms, thus responding to certain priorities identified in the mental health strategy for high-performance sport in Canada (89).

### Study limitations

The current study has some noteworthy limitations. First, the study uses self-reported data, which are less accurate than data obtained by more objective methods for assessing ED symptoms (e.g., clinical interviews). For example, athletes tend to underestimate the signs and symptoms of eating problems when completing a self-administered questionnaire (9). Second, given that the participants in this study represent a sample of convenience, we cannot speak of prevalence, as this sample may not be representative of the study population. Thus, a proportion and not a prevalence of NextGen athletes displaying drive for thinness behaviors and attitudes was presented in this study. Third, given the cross-sectional design employed, only associations between variables can be identified, making it impossible to determine whether the variables identified predispose, co-occur or result from ED symptoms in this particular population. A longitudinal study would be necessary to verify this. In addition, qualitative studies would also be required to investigate certain hypotheses proposed in the present study (e.g., impact of body esteem on EDs in NextGen athletes) and to better understand how the factors explored in this study play out in the drive for thinness and muscularity. Fourth, we observed that athletes who were “neither studying nor employed” exhibited more drive for thinness attitudes and behaviors than did those who were studying and/or employed, yet few participants were in this situation (*n* = 9). Finally, while the uniquely Francophone sample may be a strength, it may also be a limitation, and further studies would be needed to develop knowledge regarding ED symptoms in English-speaking NextGen Canadian athletes.

## Conclusion

This study highlighted the importance of taking into account both the drive for thinness and drive for muscularity behaviors and attitudes in order to obtain an accurate and comprehensive picture of EDs in francophone NextGen athletes. Several factors identified in this study (e.g., athlete status, perfectionism) appear to play a role in the development of EDs in this population. The study of other factors such as athletic identity and the coach-athlete relationship could provide insight into the development of ED symptoms within this group of athletes. Thus, further research is needed to better understand the impact of athletes’ reality on their ED symptoms.

## Data availability statement

The datasets presented in this article are not readily available because participants consented to only members of the research team having access to the data. Requests to access the datasets should be directed to VB, Veronique.Boudreault2@USherbrooke.ca.

## Ethics statement

The studies involving humans were approved by Research Ethics Committee Education and Social Sciences of the University of Sherbrooke. The studies were conducted in accordance with the local legislation and institutional requirements. Written informed consent for participation was not required from the participants or the participants’ legal guardians/next of kin because participants aged between 14 and 18 wishing to take part were asked to submit an information letter to their parents containing all the information about the project. However, it was the participants who gave their free and informed consent to take part in this study.

## Author contributions

JM: Conceptualization, Data curation, Funding acquisition, Writing – original draft, Writing – review & editing. SL: Conceptualization, Formal analysis, Methodology, Writing – original draft, Writing – review & editing. LP-F: Conceptualization, Data curation, Methodology, Writing – original draft, Writing – review & editing. VB: Conceptualization, Data curation, Funding acquisition, Project administration, Writing – review & editing. SB: Conceptualization, Data curation, Project administration, Writing – review & editing. JD: Writing – review & editing. ND-B: Writing – review & editing. SP: Writing – review & editing. AS: Writing – review & editing.
