# Supplementary material for: Personal risk factors and types of sport associated with drive for thinness and drive for muscularity in NextGen athletes
Source: Front Nutr. 2024 Jun 10;11:1392064. doi: 10.3389/fnut.2024.1392064 (PMC11194407; doi:10.3389/fnut.2024.1392064)
Supplement: Supplementary file 1 [file Table_1.docx]

Supplementary Material

*Sample description (N = 254)*

|  | Percentage (frequency) | Mean (SD) |
| --- | --- | --- |
| EAT-26 |  | 8,65 (8,99) |
| DMS-FR |  | 22,50 (7,91) |
| Athletic status      Relève      Élite | 46.5 (118)  53.5 (136) |  |
| Sex at birth      Male      Female      Intersex | 35.5 (90)  64.5 (164)  0.0 (0) |  |
| Gender identity      Man      Woman      Other gender identity | 35.4 (90)  63.4 (161)  1.2 (3) |  |
| Sexual orientation      Heterosexual      Bisexual      Homosexual      Other sexual orientation | 91.7 (233)  5.9 (15)  1.2 (3)  1.2 (3) |  |
| Ethnic or cultural group (athletes could select more than one option when applicable)     Canadian      First Nations, Inuit, Métis, Aboriginal     Latin American (Central and South America)      Afro-American (Caribbean, West Indies)      Sub-Saharan African (Gabon, Senegal, etc.)     North African (Maghreb)/Middle East      Asian (China, Japan, Laos, Philippines, etc.)      Western European (France, Spain, etc.)     Eastern European (Hungary, Romania, etc.)      Other ethnicity (specify) | 95.7 (243) 1.2 (3) 1.6 (4) 0.4 (1) 0.0 (0) 3.1 (8) 3.1 (8) 3.9 (10) 2.8 (7) 0.4 (1) |  |
| Highest level of education      Secondary School not completed/withdrawn      Secondary School in progress      Secondary School Diploma      CEGEP      Bachelor’s degree      Master’s degree      PhD | 1.2 (3)  35.8 (91)  46.1 (117)  14.6 (37)  1.2 (3) 0.8 (2)  0.4 (1) |  |
| Currently enrolled in a study program     Yes          High school          CEGEP          University      No | 92.5 (235)  29.8 (70)  54.5 (128)  15.7 (37)  7.5 (19) |  |
| Currently employed      Yes             0-5 hours/week             5-10 hours/week             10-15 hours/week             15-20 hours/week             20-25 hours/week             Over 30 hours/week      No | 48.0 (122)  18.9 (23)  23.8 (29)  25.4 (31)  16.4 (20)  4.1 (5)  11.5 (14)  52.0 (132) |  |
| Occupation      Neither in school or employed      In school or employed    In school and employed | 3.5 (9)  52.4 (133)  44.1 (112) |  |
| Type of sport      Team      Endurance      Aesthetic      Weight-category      Technical | 31.1 (79)  30.7 (78)  11.8 (30)  11.8 (30)  14.6 (37) |  |
| Age where the athlete started competing in their main sport |  | 9.29 (3.46) |
| Had to move away from the family home to train at a remote site      Yes             I was between 11 and 15 years old             I was over 16 years old      No | 32.3 (82)  31.7 (26)  56.3 (56)  66.9 (170) |  |
| Housing situation      Parents’ house      Apartment or house with roommates      Billeting      Living alone      Apartment or house with a partner      Other (e.g., in a university residence) | 72.4. (184)  13.8 (35)  6.7 (17)  3.1 (8)  2.4 (6)  1.6 (4) |  |

*Characteristics: Type of sports*

| **Type of Sport** | **Definition** | **Sports included in the current study** |
| --- | --- | --- |
| Weight-Category sport | Sports where athletes are divided by weight category to face an opponent with a similar physique to theirs. They must be below a certain weight to participate in their sports competition. | Judo (n = 8)  Boxing (n = 3)  Powerlifting (n = 8)  Karate (n = 3)  Taekwondo (n = 8) |
| Aesthetic sports | Sports where athletes are judged by an external person on the aesthetics of their performance. | Gymnastics (n = 10)  Diving (n = 3)  Synchronized swimming (n = 10)  Figure skating (n = 7) |
| Endurance sports | Sports that include a race where athletes perform for a specific period and are ranked according to their arrival at the finish line | Rowing (n = 3)  Cycling (n = 10)  Track and field (n=13)  Canoeing-kayaking (n = 7)  Swimming (n = 22)  Speed Skating (n = 9)  Alpine skiing (n = 7)  Cross-country skiing (n = 5)  Biathlon (n = 1)  Triathlon (n = 1) |
| Technical sports | Sports where athletes are judged on the technique of their sport. | Acrobatic skiing (n = 11) Tennis (n = 2) Equestrian (n = 1)  Badminton (n = 3) Fencing (n = 8) Rock climbing (n = 1) Golf (n = 3) Kite surfing (n = 1) Snowboarding (n = 2) Squash (n = 1) Archery (n = 1) Table tennis (n = 3) |
| Team sports | Sports where positions are assigned to athletes. The athletes are part of a team and compete against an opposing team on a field. | Basketball (n = 2)  Volleyball (n = 15) Football (n = 2)  Softball (n = 5) Baseball (n = 11)  Water polo (n = 4) Handball (n = 1) Hockey (n = 32) Soccer (n = 4) Ultimate frisbee (n = 1) Wheelchair basketball (n = 1) Rugby (n = 1) |

*Correlation Matrix of All Study Variables (Part 1)*

|  | 1. | 2. | 3. | 4. | 5. | 6. | 7. | 8. | 9. | 10. | 11. | 12. | 13. | 14. |
| --- | --- | --- | --- | --- | --- | --- | --- | --- | --- | --- | --- | --- | --- | --- |
| 1. Sex at birth | 1 |  |  |  |  |  |  |  |  |  |  |  |  |  |
| 2. Age | -.027 | 1 |  |  |  |  |  |  |  |  |  |  |  |  |
| 3. Athletic status | -.030 | .364 | 1 |  |  |  |  |  |  |  |  |  |  |  |
| 4. Sexual orientation | .163 | -.066 | -.007 | 1 |  |  |  |  |  |  |  |  |  |  |
| 5. Highest level of education | .004 | .809 | .269 | -.057 | 1 |  |  |  |  |  |  |  |  |  |
| 6. Satisfaction with current athletic performance | -.227 | -.042 | .011 | -.108 | -.106 | 1 |  |  |  |  |  |  |  |  |
| 7. Perceived athletic competence | -.259 | -.081 | .025 | -.152 | -.088 | -.568 | 1 |  |  |  |  |  |  |  |
| 8. Global sport-state self-esteem | -.272 | -.066 | .019 | -.143 | -.110 | .914 | .853 | 1 |  |  |  |  |  |  |
| 9. Perfectionist aspirations during training | -.039 | -.171 | -.070 | .010 | -.145 | -.179 | .011 | -.108 | 1 |  |  |  |  |  |
| 10. Negative reactions to non-perfect performance during training | .105 | -.028 | .030 | .149 | -.006 | -.549 | -.275 | -.484 | .522 | 1 |  |  |  |  |
| 11. Pressure from parents to be perfect | .080 | -.130 | .011 | .120 | -.073 | -.272 | -.002 | -.173 | .096 | .321 | 1 |  |  |  |
| 12. Striving for perfection | -.042 | -.187 | -.065 | -.004 | -.164 | -.157 | .029 | -.086 | .987 | .489 | .091 | 1 |  |  |
| 13. Negative reactions to imperfection | .141 | -.003 | .020 | .145 | .007 | -.542 | -.286 | -.485 | .492 | .978 | .304 | .458 | 1 |  |
| 14. Global perfectionism | .053 | -.116 | -.028 | .079 | -.096 | -.400 | -.143 | -.324 | .878 | .846 | .226 | .867 | .840 | 1 |

Sex at birth (0 = Male, 1 = Female), Athletic status (0=relève, 1=elite), Sexual orientation (0=heterosexual, 1= sexual minority), Remoteness from family for training (0=no, 1=yes). The analyses include dummy coded variables.^1^ The first variable is the type of commitment the participants had in parallel with their sport: being neither in school nor employed (1= being neither in school nor employed, 0=other commitment), being in school or employed (1= being in school or employed, 0=other commitment), and being in school and employed (1= being in school and employed, 0=other commitment).^2^ The second variable is the category of sport practiced by participants: team sport (1=team sport, 0= other sport), endurance sport (1=endurance sport, 0=other sport), aesthetic sport (1=aesthetic sport, 0=other sport), weight-category sport (1= weight-category sport, 0=other sport), and technical sport (1=technical sport, 0=other sport). **p* .05, ***p* .01

*Correlation Matrix of All Study Variables (Part 2)*

|  | 1. | 2. | 3. | 4. | 5. | 6. | 7. | 8. | 9. | 10. | 11. | 12. | 13. | 14. |
| --- | --- | --- | --- | --- | --- | --- | --- | --- | --- | --- | --- | --- | --- | --- |
| 15.  Being neither in school nor employed^1^ | .053 | -.080 | -.078 | .020 | -.138 | -.046 | -.071 | -.064 | .024 | .058 | .001 | .020 | .040 | .035 |
| 16.  Being in school or employed^1^ | -.130 | -.169 | -.019 | -.114 | -.113 | .051 | .005 | .035 | .128 | .077 | .025 | .126 | .051 | .105 |
| 17. Being in school and employed^1^ | .111 | .199 | .048 | .108 | .165 | -.034 | .021 | -.011 | -.138 | -.098 | -.025 | -.134 | -.066 | -.119 |
| 18.  Age of start of competition in main sport | .058 | .315 | -.104 | .095 | .323 | -.090 | -.131 | -.121 | -.065 | -.034 | .010 | -.068 | -.027 | -.056 |
| 19.  Remoteness from family for training | .065 | -.098 | .084 | -.027 | -.087 | .049 | .069 | .065 | .090 | .054 | -.018 | .093 | .044 | .082 |
| 20.  Team sport^2^ | -.107 | -.067 | .285 | .169 | -.135 | .001 | .102 | .051 | .103 | .060 | .136 | .102 | .054 | .093 |
| 21.  Endurance sport^2^ | -.042 | .210 | -.064 | -.138 | .250 | .053 | -.018 | .025 | -.147 | -.093 | -.098 | -.161 | -.086 | -.146 |
| 22. Aesthetic sport^2^ | .144 | -.154 | -.026 | .023 | -.128 | -.016 | -.124 | -.071 | .078 | .057 | -.107 | .106 | .062 | .099 |
| 23.  Weight-category sport^2^ | .042 | .071 | -.075 | -.066 | .076 | .010 | -.003 | .005 | .008 | -.056 | .025 | -.003 | -.038 | -.024 |
| 24.  Technical sport^2^ | .026 | -.110 | -.197 | -.002 | -.101 | -.065 | .006 | -.038 | -.021 | .042 | .025 | -.017 | .020 | .000 |
| 25. Drive for muscularity | -.244 | .062 | .162 | .018 | .119 | -.164 | .016 | -.096 | .239 | .203 | .062 | .226 | .198 | .249 |
| 26. Drive for thinness | .095 | -.080 | .013 | .055 | -.069 | -.179 | -.054 | -.140 | .264 | .347 | .102 | .261 | .314 | .335 |

Sex at birth (0 = Male, 1 = Female), Athletic status (0=relève, 1=elite), Sexual orientation (0=heterosexual, 1= sexual minority), Remoteness from family for training (0=no, 1=yes). The analyses include dummy coded variables.^1^ The first variable is the type of commitment the participants had in parallel with their sport: being neither in school nor employed (1= being neither in school nor employed, 0=other commitment), being in school or employed (1= being in school or employed, 0=other commitment), and being in school and employed (1= being in school and employed, 0=other commitment).^2^ The second variable is the category of sport practiced by participants: team sport (1=team sport, 0= other sport), endurance sport (1=endurance sport, 0=other sport), aesthetic sport (1=aesthetic sport, 0=other sport), weight-category sport (1= weight-category sport, 0=other sport), and technical sport (1=technical sport, 0=other sport). **p* .05, ***p* .01

*Correlation Matrix of All Study Variables (Part 3)*

|  | 15. | 16. | 17. | 18. | 19. | 20. | 21. | 22. | 23. | 24. | 25. | 26. |
| --- | --- | --- | --- | --- | --- | --- | --- | --- | --- | --- | --- | --- |
| 15. Being in school or employed | 1 |  |  |  |  |  |  |  |  |  |  |  |
| 16.  Being in school and employed | -.931 | 1 |  |  |  |  |  |  |  |  |  |  |
| 17.  Not in school nor employed | -.201 | -.170 | 1 |  |  |  |  |  |  |  |  |  |
| 18.  Age of start of competition in main sport | -.036 | .028 | .021 | 1 |  |  |  |  |  |  |  |  |
| 19.  Remoteness from family for training | .085 | -.079 | -.018 | -.072 | 1 |  |  |  |  |  |  |  |
| 20.  Team sport | .045 | -.049 | .009 | -.171 | .038 | 1 |  |  |  |  |  |  |
| 21.  Endurance sport | -.014 | .028 | -.035 | .178 | -.059 | -.447 | 1 |  |  |  |  |  |
| 22. Aesthetic sport | -.042 | .019 | .062 | -.175 | -.033 | -.246 | -.244 | 1 |  |  |  |  |
| 23.  Weight-category sport | -.066 | .068 | -.004 | .051 | -.033 | -.246 | -.244 | -.134 | 1 |  |  |  |
| 24.  Technical sport | .059 | -.052 | -.019 | .105 | .089 | -.277 | -.275 | -.151 | -.151 | 1 |  |  |
| 25. Drive for muscularity | -.009 | .036 | -.072 | .047 | -.035 | .125 | .092 | -.204 | -.022 | -.079 | 1 |  |
| 26. Drive for thinness | .081 | -.140 | .157 | -.076 | -.031 | .027 | -.089 | .040 | .059 | -.010 | .247 | 1 |

Sex at birth (0 = Male, 1 = Female), Athletic status (0=relève, 1=elite), Sexual orientation (0=heterosexual, 1= sexual minority), Remoteness from family for training (0=no, 1=yes). The analyses include dummy coded variables.^1^ The first variable is the type of commitment the participants had in parallel with their sport: being neither in school nor employed (1= being neither in school nor employed, 0=other commitment), being in school or employed (1= being in school or employed, 0=other commitment), and being in school and employed (1= being in school and employed, 0=other commitment).^2^ The second variable is the category of sport practiced by participants: team sport (1=team sport, 0= other sport), endurance sport (1=endurance sport, 0=other sport), aesthetic sport (1=aesthetic sport, 0=other sport), weight-category sport (1= weight-category sport, 0=other sport), and technical sport (1=technical sport, 0=other sport). **p* .05, ***p* .01
